# The Protective Role of Decorin in Hepatic Metastasis of Colorectal Carcinoma

**DOI:** 10.3390/biom10081199

**Published:** 2020-08-18

**Authors:** Andrea Reszegi, Zsolt Horváth, Katalin Karászi, Eszter Regős, Victoria Postniková, Péter Tátrai, András Kiss, Zsuzsa Schaff, Ilona Kovalszky, Kornélia Baghy

**Affiliations:** 1 1st Department of Pathology and Experimental Cancer Research, Semmelweis University, Üllői Street 26, H-1085 Budapest, Hungary; areszegi2@gmail.com (A.R.); opponent01@gmail.com (Z.H.); tika0604@gmail.com (K.K.); eszter.regos.88@gmail.com (E.R.); postnikova.victoria@gmail.com (V.P.); kovalszky.ilona@med.semmelweis-univ.hu (I.K.); 2 Solvo Biotechnology, H-1117 Budapest, Hungary; tatrai@solvo.com; 3 2nd Department of Pathology, Semmelweis University, H-1091 Budapest, Hungary; kiss.andras@med.semmelweis-univ.hu (A.K.); schaff.zsuzsa@med.semmelweis-univ.hu (Z.S.)

**Keywords:** decorin, ECM, colorectal carcinoma, RTK, signaling, liver metastasis

## Abstract

Decorin, the prototype member of the small leucine-rich proteoglycan gene family of extracellular matrix (ECM) proteins, acts as a powerful tumor suppressor by inducing the p21^Waf1/Cip1^ cyclin-dependent kinase inhibitor, as well as through its ability to directly bind and block the action of several tyrosine kinase receptors. Our previous studies suggested that the lack of decorin promotes hepatic carcinogenesis in mice. Based on this, we set out to investigate whether excess decorin may protect against the liver metastases of colon carcinoma. We also analyzed the effect of decorin in tissue microarrays of human colon carcinoma liver metastasis and examined whether the tumor cells can directly influence the decorin production of myofibroblasts. In humans, low levels of decorin in the liver facilitated the development of colon carcinoma metastases in proportion with more aggressive phenotypes, indicating a possible antitumor action of the proteoglycan. In vitro, colon carcinoma cells inhibited decorin expression in LX2 hepatic stellate cells. Moreover, liver-targeted decorin delivery in mice effectively attenuated metastasis formation of colon cancer. Overexpressed decorin reduced the activity of multiple receptor tyrosine kinases (RTKs) including the epidermal growth factor receptor (EGFR), an important player in colorectal cancer (CRC) pathogenesis. Downstream of that, we observed weakened signaling of ERK1/2, PLCγ, Akt/mTOR, STAT and c-Jun pathways, while p38 MAPK/MSK/CREB and AMPK were upregulated culminating in enhanced p53 function. In conclusion, decorin may effectively inhibit metastatic tumor formation in the liver.

## 1. Introduction

Colorectal cancer (CRC) is the second leading cause of cancer death worldwide [1,2]. The risk of development of liver metastasis highly depends on the site of the primary tumor. CRC is one of the primary malignancies that most frequently invade the liver [3]. Beside resection, liver transplantation may be considered as a radical surgical treatment option for patients with metastatic liver cancer who are eligible for this procedure [3,4]. With the rise of targeted therapies, epidermal growth factor receptor (EGFR) inhibitors were introduced and since then have been successfully applied to patients with CRC [5].

The extracellular matrix (ECM) is a dynamic network found in all tissues and organs. It comprises proteoglycans (PG), glycosaminoglycans (GAG), fibronectin, collagens, laminins, elastin, hyaluronic acid (HA), and glycoproteins, as well as growth factors, cytokines and chemokines [6]. With its well-organized structure, the ECM provides a physical scaffold for the adhesion, differentiation, migration and proliferation of cells, and regulates cellular signaling [7]. In chronic liver diseases, the ECM undergoes profound molecular remodeling that affects both the structure and abundance of ECM proteins [8]. Cancer progression in the liver is characterized by excessive deposition of ECM proteins synthesized by activated hepatic stellate cells (HSCs) [8,9]. The thorough investigation of these ECM alterations is key to a better understanding of tumor progression in the liver and may lead to the identification of new therapeutic targets [10]. These aims have fueled several lines of recent research into the relationship between the tumor and ECM.

Decorin (DCN) is the prototype member of the small leucine-rich proteoglycan (SLRP) gene family of ECM proteins [11]. DCN contains 12 leucine-rich repeats and carries a single GAG chain of chondroitin or dermatan sulfate attached to its 42-kDa protein core [12]. Under normal conditions, decorin is produced by fibroblasts, myofibroblasts, smooth muscle cells and endothelial cells [11]. This proteoglycan is known for its high-affinity interaction with collagen fibers [12] and plays important roles in several physiological and pathological processes such as collagen fibrillogenesis [13,14], myocardial infarction [15], muscular development [16], wound healing [17], kidney [18] and liver fibrosis [19], regulation of autophagy and inflammation [20], as well as tumor growth [21], angiogenesis, and migration [22]. In the normal liver, decorin is located around central veins and in the portal tracts. During malignant transformation, together with other matrix proteins the amount of decorin increases significantly in the connective tissue septa [23,24,25]. Decorin acts as a powerful tumor suppressor by inducing the p21^WAF1/CIP1^ cyclin-dependent kinase inhibitor [26], as well as through its ability to directly bind and block the action of several receptor tyrosine kinases (RTKs) such as EGFR [27,28], the hepatocyte growth factor receptor Met [29], vascular endothelial growth factor receptor (VEGFR) [30], and insulin-like growth factor-1 receptor (IGF-1R) [31].

In our previous studies, we have shown in two in vivo mouse models that lack of decorin promotes primary hepatocarcinogenesis [32], while excess decorin counteracts the process [33]. These findings implied a protective role for decorin against liver cancer formation. Based on these results, we designed a new set of experiments to investigate whether decorin also protects against liver metastases of colon carcinoma. We first determined changes in the level of decorin in the liver metastases of human colon carcinoma and examined whether the tumor cells may directly influence the production of decorin by myofibroblasts. Subsequently, we investigated whether excessive decorin production may attenuate metastasis formation in the liver and whether decorin may act as a physiological inhibitor of RTKs in liver metastases.

## 2. Materials and Methods

### 2.1. Tissue Microarray (TMA)

In a collaboration between the 1st and 2nd Departments of Pathology of the Semmelweis University, we have collected archived biopsy samples from 30 patients with liver metastases of CRC. Approval was obtained from the Regional and Institutional Committee of Science and Research Ethics (TUKEB permit number: 95/1999). Tissue microarrays were assembled from tissue cores containing liver metastases and surrounding non-tumorous liver tissue as well as the primary tumor and normal colon tissue of each patient. Stromal reaction was categorized as immature, intermediate and mature in primary CRC tumor samples [34], and as desmolastic or replacement type in liver metastases [35]. The list of the biopsy samples with clinicopathological data is provided in Supplementary Table S1. The TMA block was sectioned, and slides were counterstained with hematoxylin and immunostained for decorin. α-Smooth muscle actin (SMA) staining of surrounding liver samples was performed to describe stroma abundance. Decorin and SMA staining intensities were analyzed and scored by the Pannoramic Viewer software TMA module (3D Histech Ltd., Budapest, Hungary) by two independent pathologists. The final score was determined by averaging the scores (Supplementary Table S2). The correlation analysis between decorin and SMA staining revealed that decorin score is independent from the amount of stroma in the liver samples (Supplementary Figure S1).

### 2.2. Plasmid Preparation

The pLIVE expression vector was applied according to the manufacturer’s guide (Liver In Vivo Expression) (Mirus Bio, Madison, WI, USA). The vector is driven by a liver-specific chimeric promoter composed of the mouse α-fetoprotein enhancer II and the minimal mouse albumin promoter. Full-length cDNA of the human decorin (DCN) gene inserted into the pGEM-1 expression vector was subcloned in the pLIVE vector using BamHI and XhoI restriction sites. The insertion was confirmed by DNA sequencing. Plasmid DNA was amplified in Escherichia coli DH5α cells, isolated by alkaline lysis, and purified using the Qiagen plasmid midi kit according to the manufacturer’s instructions (Qiagen, Valencia, CA, USA). The quality and quantity of the plasmid DNA was analyzed by restriction endonuclease digestion, agarose gel electrophoresis, and absorbance at 260/280 nm by ND-1000 spectrophotometer (NanoDrop Technologies, Wilmington, DE, USA).

### 2.3. Animal Experiments

All animal studies were performed according to the ethical standards of the Animal Health Care and Control Institute, Csongrád County, Hungary (ethical license: XVI/03047-2/2008).

Thirty two-month-old male C57BL/6 mice were used for the experiments. Plasmid DNA (pLIVE-DCN or pLIVE-0 (control)) was injected by hydrodynamic gene delivery technique according to the manufacturer’s user guide (Mirus Bio LLC, Madison, WI, USA). In brief, 15 μg of high quality/purity plasmid DNA was prepared in 2 mL of pharmaceutical-grade saline solution at room temperature. Mice were anesthetized, and the lateral tail vein was accessed using a 27-gauge needle (according Mirus Bio LLC). Administration of the solution was performed within 4–7 s, at a constant rate, without extravasation. Each group contained 15 animals. In vivo transfection was followed by a 3-day recovery period prior to tumor cell inoculation.

The c38 CRC cell line was maintained by serial subcutaneous transplantations in C57BL/6 mice. Inbred C57BL/6 mice from our institute were used throughout the studies. Tumor tissue was cut into small pieces in serum-free RPMI-1640 medium and digested by collagenase I (Sigma, St. Louis, MO, USA; 7 mg collagenase/10 mL medium) at 37 °C for 30 min; the suspension was filtered through 4-fold gauze. After centrifugation and washing, the viability of the tumor cells was determined by the Trypan blue exclusion test. To obtain liver metastases, 2 × 10^5^ tumor cells were injected into the spleen of mice anesthetized by sodium pentobarbital (Nembutal, 70 mg/kg). Mice were sacrificed 21 days after tumor cell inoculation. Body and liver weights of each animal were recorded, and metastases were counted. Half of each sample was embedded in paraffin (FFPE samples) and the other half was frozen for further experiments.

### 2.4. Immunostaining

Immunostaining was performed on formalin-fixed paraffin-embedded (FFPE) human tissue microarray sections and FFPE sections of mouse liver according to standard protocols [32]. Anti-Decorin (Sigma-Aldrich St. Louis, MO, #HPA003315, 1:1000) and α-smooth muscle actin (SMA) (#M0851, 1:50, Dako Agilent, Santa Clara, CA, USA) were detected using the appropriate secondary antibodies (#P0448 Anti-Rabbit immunglobulin/HRP for decorin and anti-mouse immungrobulin/HRP #P0447 for SMA, Dako Agilent, 1:2000).

### 2.5. Tissue Culture and Reagents

LX2 human hepatic stellate cell (a kind gift from Dr. Scott Friedman) and HT29 human colorectal adenocarcinoma cell lines (purchased from the ATCC (Manassas, VA, USA, #HTB-38)) were cultured in RPMI-1640 (Sigma-Aldrich, St Louis, MO, USA), supplemented with 10% [*v*/*v*] fetal bovine serum (FBS, Sigma Aldrich), and 40 mg/mL gentamicin (Sigma Aldrich) at 37 °C in a humidified atmosphere with 5% CO_2_.

To obtain cell-conditioned medium (CM), the HT29 cell line was grown to 80% confluence, and the medium was replaced with fresh RPMI-1640. The CM was collected 16 h later. For treatment with the CM, LX2 cells were grown to 80% confluency in a 6-well plate. Subsequently, HT29-CM was added to the cells for 24 h, then the cells were serum-starved overnight in FBS-free RPMI-1640. LX2 cells with FBS-free medium served as control. After treatment, LX2 cells and the cell culture supernatants were collected for protein and RNA studies.

### 2.6. Western Blot, Dot Blot and Array Analysis

Arrays (Proteome Profiler Phospho-Kinase Array Kit, Phospho-Receptor Tyrosine Kinase Array Kit (R&D Systems, Inc., Minneapolis, MN, USA)), western blot and dot blot analyses were performed on frozen liver samples (three livers of each experimental group were pooled) and cell lysates. All analyses were done as previously described [32,36] and array signals were developed according to the manufacturer’s instructions.

For dot blot, 200 µL cell culture media and for western blot, 20 μg of total proteins were applied on PVDF membrane. The following primary antibodies were used: Decorin (Sigma-Aldrich, #HPA003315, 1:1000), p44/42 MAPK (Erk1/2) (Cell Signaling, #4695, 1:1000), phospho-p44/42 MAPK (Erk1/2) (Cell Signaling, #4377, 1:1000), GSK-3α/β (Cell Signaling, #5676, 1:1000), phospho-GSK-3α/β (Cell Signaling, #9331, 1:1000), Akt (pan) (Cell Signaling, #4691, 1:1000), Phospho-Akt (Thr308) (Cell Signaling, #2965, 1:1000), β-Catenin (Cell Signaling, #9562, 1:1000), p27^Kip1^ (Biosource, #AHZ0458, 1:500), and β-Actin (Sigma-Aldrich, #A2228, 1:5000). The primary antibodies were detected using Anti-Rabbit immunglobulin/HRP (DakoCytomation, #P0448, 1:2000) and Anti-Mouse immunglobulin/HRP (DakoCytomation, #P0447, 1:2000) secondary antibodies. For Array kits, 1000 μg of protein in 2000 μL lysate was applied to the membrane. Signals were detected by enhanced chemiluminescence (SuperSignal West Pico Chemiluminescent Substrate Kit (Thermo Fisher Scientific Inc., Waltham, MA, USA)) and visualized on iBright FL1500 Imaging System (Thermo Fisher Scientific Inc.).

### 2.7. Real-Time qPCR

For RT-qPCR, total RNA was isolated from treated LX2 cells and performed according to the manufacturer’s standard protocols (RNAeasy Mini kit (Qiagen, Hilden, Germany); High Capacity cDNA kit (Invitrogen, Carlsbad, CA, USA)), as previously described [32]. Real-time PCR was performed using TaqMan Gene Expression Assays for human decorin (DCN, Assay ID: Hs00370383_m1, Life Technologies, by ThermoFisher, Waltham, MA, USA) and 18S ribosomal RNA (Part No.:4319413E, Life Technologies). Samples were run in duplicate in 20 μL reaction volume containing 50 ng cDNA using TaqMan Universal PCR Master Mix (Part No.:4324018, Applied Biosystems). Results were determined as threshold cycle values. Relative expression levels were calculated using the 2^−ΔΔC^_T_ method.

### 2.8. Enzyme-Linked Immunosorbent Assay (ELISA)

Human decorin levels from the serum of mice were quantified by sandwich enzyme-linked immunosorbent assay, using the Human Decorin ELISA Kit from Sigma-Aldrich (Cat.No. #RAB0140 Sigma, MO, USA), following the manufacturer’s user guide. Each sample was analyzed in duplicate. ELISA plates were read at 570 nm with a Labsystems Multiscan MS 352 (Thermo Labsystems, Helsinki, Finland) plate reader.

### 2.9. Statistical Analysis

All data are representative of three independent experiments. Mean values and SD were calculated by GraphPad Prism 4.03 software (Graphpad Software Inc., La Jolla, CA, USA). Statistical analysis was performed using D’Agostino and Pearson’s omnibus normality test; Mann-Whitney or Students’ *t*-tests were used for statistical calculations of significance. *p* value < 0.05 was declared as statistically significant.

## 3. Results

### 3.1. Decorin Expression Decreased in Liver Metastases of Human CRC

**Figure 1:**
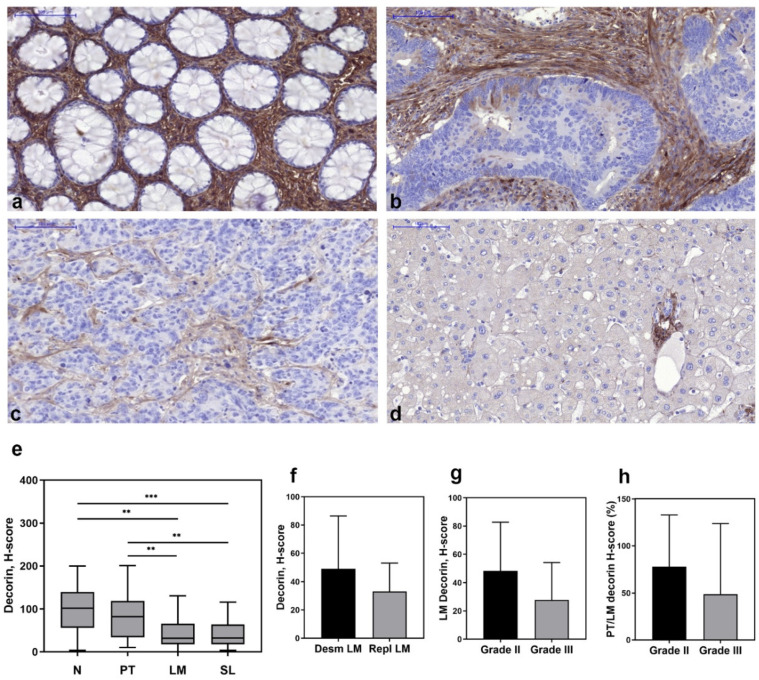
Immunohistochemistry of decorin in normal colon (**a**), colorectal cancer (**b**), liver metastasis (**c**), and surrounding liver tissue (**d**). Decorin expression in primary tumor stroma is usually high (**b**); however, liver metastasis of the same tumor often displays a reduced amount of the proteoglycan (**c**). Bar charts represent the amount of decorin (*n* = 30) (**e**). Decorin level is lower in the replacement type of metastases than those of desmoplastic ones (**f**). Metastases of grade III tumors are characterized with lower decorin expression compared to grade II CRC (**g**). Grade III metastases contain less decorin in their paired primary tumors than those of grade II shown by relative decorin levels (**h**). N = Normal colon, PT = primary tumor, LM = liver metastasis, SL = surrounding liver, Desm = desmplastic type of LM (*n* = 20), Repl. = replacement type of LM (*n* = 10). Grade II: *n* = 23, grade III: *n* = 6. Scale bar = 100 µm. Data are presented as mean (S.D.). ** *p* value < 0.01; *** *p* value < 0.001.

Human tissue microarrays were assembled from biopsy samples of normal colon tissue, primary tumor, liver metastasis and surrounding liver tissue from each patient, and immunostaining for decorin was performed (Figure 1a–d, Table S2).

The highest decorin levels were observed in the stroma of normal colon tissue (Figure 1a,e). In the stroma of primary colon tumors, while decorin was still abundant, its expression was reduced compared to the normal tissue (Figure 1b,e). The lowest amount of decorin, significantly less than in the normal colon and primary tumors, was detected in liver metastases (*p* value < 0.01) (Figure 1c,e). The level of decorin expression in liver metastases was similar to that seen in peritumoral liver tissue (Figure 1e). Decorin level did not correlate with the type of desmoplastic reaction in primary tumors. However, we measure decreased decorin amounts in metastases with the more aggressive replacement growing pattern compared to desmoplastic ones (Figure 1f). In line with that, grade III metastases contained less decorin than those of grade III tumors when measuring its absolute level (Figure 1g), as well as its relative level compared to the primary tumor (Figure 1h). Based on these observations, we speculate that decreased expression of decorin in liver metastases compared to the primary tumors may reflect the aggressiveness of the metastatic tumor.

### 3.2. Decorin Expression of Myofibroblasts Is Inhibited by Tumor Cells In Vitro

To test whether tumor cells are capable of directly influencing the decorin production of myofibroblasts, LX2 human stellate cells were exposed to CM of HT29 human colon adenocarcinoma cells.

**Figure 2:**
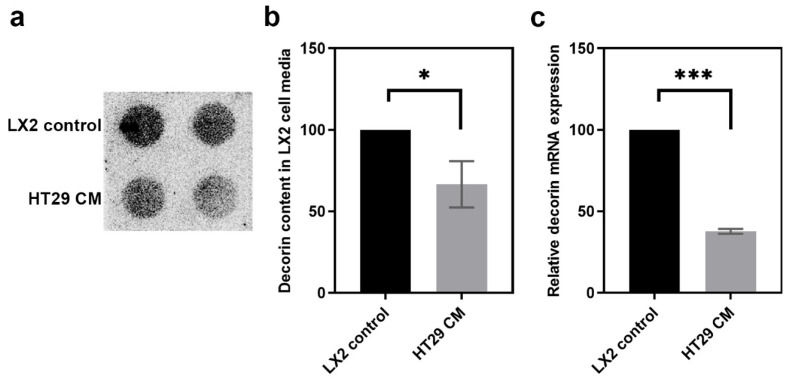
Decorin production in LX2 stellate cells upon exposure to HT29 human colon adenocarcinoma cell medium. Dot blot analysis of decorin content in LX2 cell media (**a**) and its quantification (**b**). Determination of decorin mRNA levels (**c**). CM = conditioned medium. Data are shown as mean (S.D.) *** *p* value < 0.001; * *p* value < 0.05.

Significantly less decorin was observed in the media of LX2 cells when HT29-CM was applied to them (*p* value < 0.05) (Figure 2a,b). The downregulation occurred at the transcriptional level, as the decorin mRNA level was significantly reduced in LX2 cells exposed to HT29-CM (*p* value < 0.001) (Figure 2c).

These results correlated well with our observations on human CRC tissue samples, indicating that tumor cells reduce the production of decorin by myofibroblasts in the liver, which supports a tumor-suppressive role for decorin.

### 3.3. Overexpressed Decorin Reduces Tumor Formation in an Experimental Mouse Model

Our previous experiments have confirmed that decorin deficiency promotes the formation of primary liver tumors in decorin gene knockout (Dcn^−/−^) mice [32]. Based on these results, we designed an in vivo colon carcinoma liver metastasis model, where c38 colon carcinoma cells were injected into the spleen and allowed to colonize the liver. Targeted transfection of human decorin into the liver was conducted using the pLIVE vector and hydrodynamic gene delivery as described previously.

**Figure 3:**
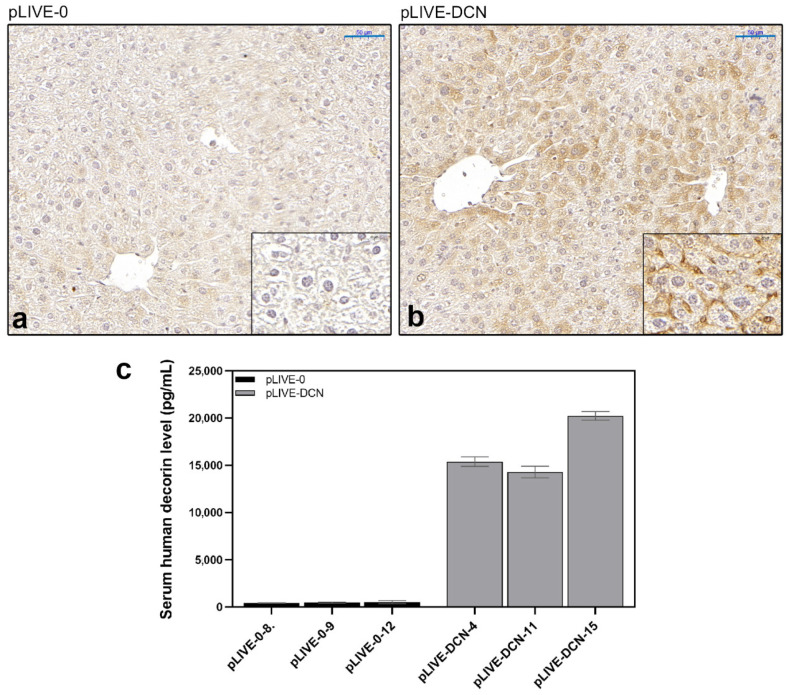
Expression of human decorin in the liver of mock-transfected (pLIVE-0) (**a**) and decorin-transfected (pLIVE-DCN) (**b**) groups. Strong immunopositivity was detected around the central veins and the portal tracts of decorin-transfected livers. (**c**) Human decorin ELISA from sera of control (pLIVE-0) and decorin-transfected (pLIVE-DCN) groups following c38 injection. The absolute proteoglycan level is marked on the *y*-axis (pg/mL) and individual mice are shown on the *x*-axis (*n* = 3 in both groups). Data are displayed as mean ± SD. Scale bar = 50 μm.

Elevated decorin expression following transfection was confirmed by immunohistochemistry and enzyme-linked immunosorbent assay. By immunostaining specific for human decorin, strong positivity was detected around the central veins and in the sinusoids in decorin-transfected livers (Figure 3a,b). Human decorin was also measured in the sera of mice, and high decorin levels were detected in the serum of decorin-transfected animals (Figure 3c). These results confirmed that the hydrodynamic gene delivery was successful and decorin was actively produced in the transfected mice.

**Figure 4:**
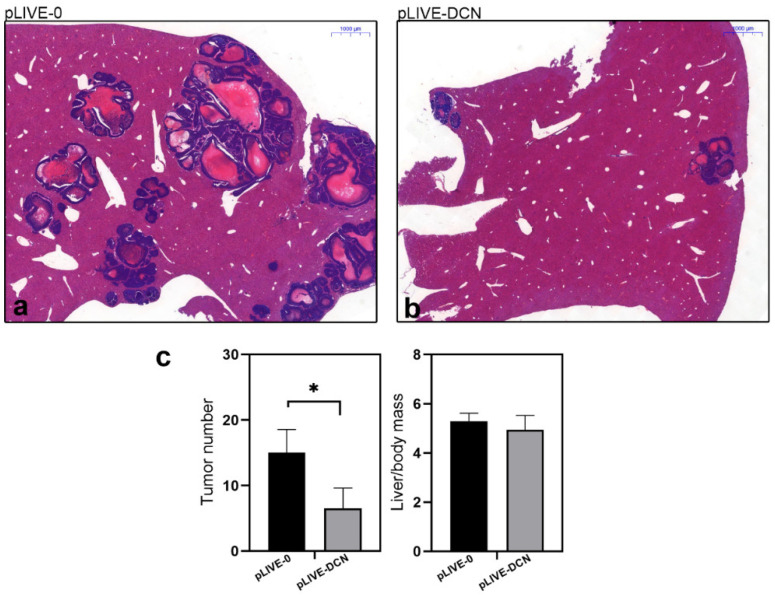
Representative histological images of hematoxylin and eosin-stained control (pLIVE-0) (**a**), and decorin expressing (pLIVE-DCN) (**b**) liver tissues after inoculation of c38 tumor cells. Bar charts (**c**) represent the ratios of tumor-bearing mice in experimental groups of control and DCN expressing groups. In livers overexpressing decorin, a reduced number of liver metastases was observed in parallel with lower liver mass/body ratio. Scale bar = 1000 µm. * *p* value < 0.05.

Three weeks after the inoculation of colon carcinoma cells, metastases appeared in the liver that maintained the phenotype of colon carcinoma as seen in hematoxylin and eosin-stained sections (Figure 4a). We observed a significant 63% reduction (*p* value < 0.05) in the number of metastases in livers overexpressing decorin, in parallel with lower liver mass/body mass ratio, indicating decreased tumor burden of the organ (Figure 4).

### 3.4. Signaling Pathways Affected by Decorin in Mouse Liver Metastases

**Figure 5:**
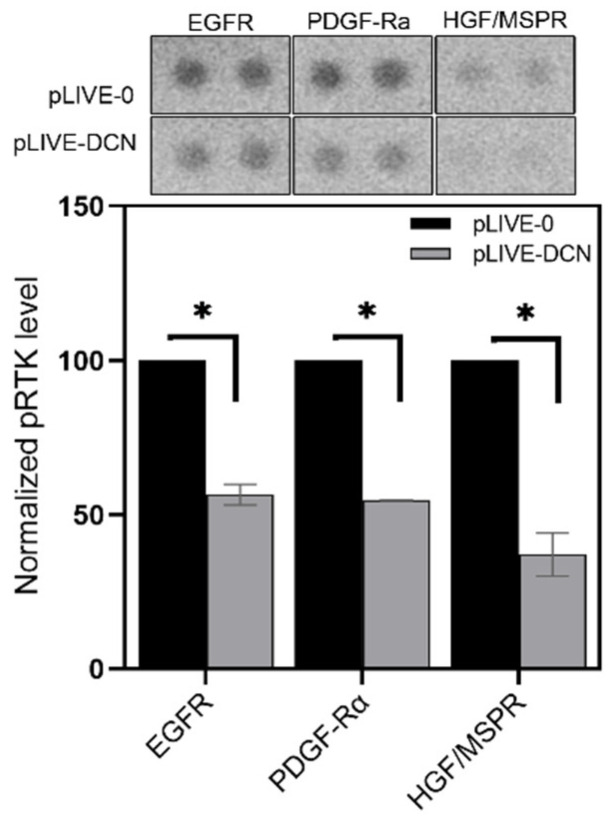
Phosphorylation of epidermal growth factor receptor (EGFR), MSPR and PDGFRα receptors in mock-transfected vs. DCN-transfected livers. Overexpression of decorin reduced the levels of active receptor tyrosine kinases (RTKs) in the DCN-transfected group. Data are shown as mean ± SD. * *p* value < 0.05.

Several studies indicated that decorin acts as an endogenous pan-RTK inhibitor. In our decorin-transfected group, excess decorin reduced the phosphorylation levels of several RTKs (Figure 5), measured by Proteome Profiler Mouse Phospho-RTK Array Kit. After c38 tumor cell inoculation, significantly less active EGFR, PDGF-Rα and HGF/MSPR receptors were detected in the livers of pLIVE-DCN animals compared to pLIVE-0 mice (*p* value < 0.05) (Figure 5). Notably, we observed a 43%, 45% and 63% reduction in phosphorylation of EGFR, PDGF-Rα and HGF/MSPR in DCN overexpressed groups, respectively (Figure 5).

**Figure 6:**
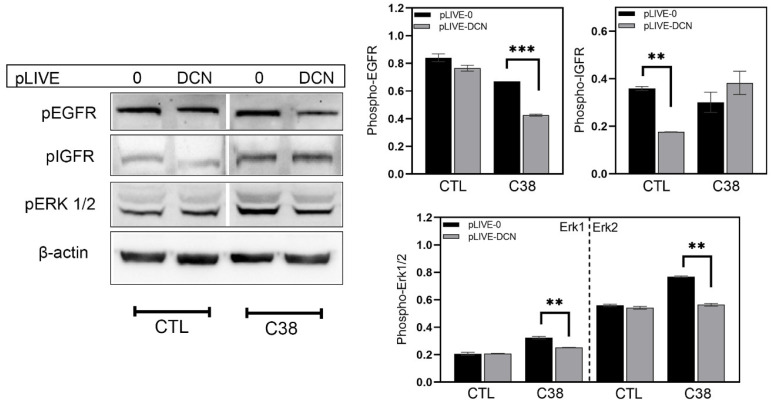
Western blot analysis of different signal transduction pathways. Bar charts illustrate the relative level of different proteins in wild type and c38-injected groups. β-actin was used as a housekeeping protein. All data are indicated as mean ± SD. ** *p* value < 0.01; *** *p* value < 0.001.

Since in our earlier experimental primary hepatocarcinogenesis model decorin was shown to induce IGFR activity [33], changes in the level of phospho-IGF-1R (pIGFR), along with pEGFR and pErk1/2, were assessed by Western blot analysis. In the tumor-free livers of sham-inoculated mice, the delivery of human decorin significantly reduced the level of pIGFR but caused no change in pEGFR or pErk1/2 (Figure 6). In the metastasis-bearing livers of c38-inoculated mice, on the other hand, transfection with pLIVE-DCN did not significantly affect pIGFR but markedly downregulated pEGFR and reduced pErk1/2 by 22% and 27%, respectively, compared to transfection with pLIVE-0 (*p* value < 0.01) (Figure 6).

**Figure 7:**
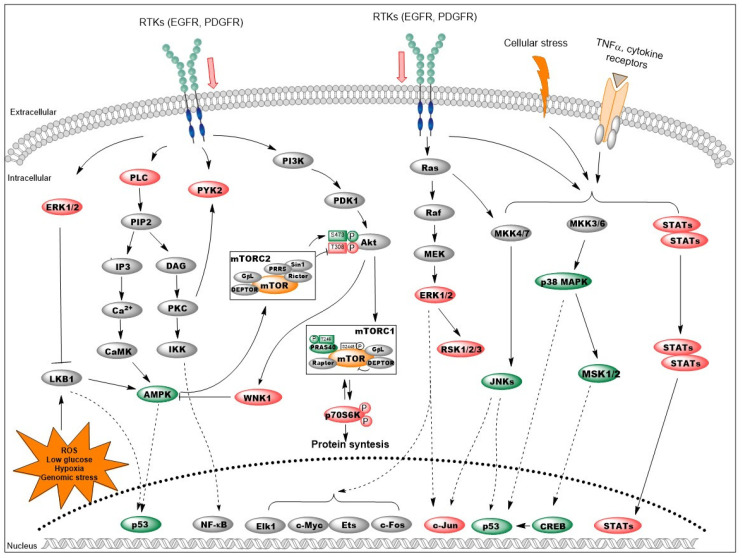
Changes in the signaling network of colorectal cancer (CRC) liver metastases upon decorin delivery. Liver-specific transfection with the DCN gene inhibited several RTKs with a concomitant decrease in the activity of the MAPK/ERK and Akt/mTOR/p70S6K pathways. Increased AMPK (possibly via LKB1) and p38/MSK/CREB signaling resulted in elevated phospho-p53 levels; stabilization of this key tumor suppressor may result in a cell cycle blockade. Inhibition of STATs and c-Jun proteins also contributes to the antitumor action of decorin. Color code: red: decreased; green: increased; orange: no change; grey: not examined. Additional details are included in the text.

Next, using a phosphokinase array we interrogated the decorin-induced changes in common signaling pathways that may have resulted in attenuated metastasis formation in the liver. Results are summarized in Table 1 and Figure 7. In general, decorin delivery suppressed the activity of most major signaling pathways. Downstream of RTKs, decorin-overexpressing animals displayed inhibition of Ras/MAPK signaling (marked by decreased ERK1/2 and RSK1/2), and attenuation of the Akt/mTOR pathway indicated by decreased pAkt(T308) and phospho-p70S6K. Concordant with decreased Akt activity, downregulation of WNK1 was also detected in the pLIVE-DCN group. The levels of most phosphorylated STAT proteins and c-Jun were lowered as well, along with reduced amounts of β-catenin, an important signaling protein in CRC and a known target of decorin treatment.

Conversely, increased activity of p38 MAPK and its downstream effectors such as MSK1/2 and CREB were detected upon decorin delivery. Overexpressed decorin raised the levels of phospho-AMPK and phospho-p53 as well. JNK proteins were also enhanced by decorin, although these alterations fell short of statistical significance.

Decorin transfection resulted in changes of two non-receptor tyrosine kinases, Fgr and PYK2, as well as of Src, all participating in the regulation of immune response and cytoskeleton remodeling. Hsp27 and Hsp60 levels were also affected.

Overall, these results suggest that the protective effect of decorin against CRC liver metastases may hinge on its blocking of RTKs.

## 4. Discussion

Decorin is a prominent member of the small leucine-rich ECM proteoglycan gene family [11]. Decorin is already well known for its powerful tumor suppressor activity, exerted via induction of the p21^Waf1/Cip1^ cyclin-dependent kinase inhibitor [26] and through its ability to directly bind to and block the action of several RTKs [27,28,29,30,31].

The expression of decorin is markedly decreased in several malignancies, such as cancers of the bladder, breast, and colon [37,38,39]. On the other hand, delivery or induced expression of decorin in different carcinomas has been demonstrated to suppress malignant behavior [40,41,42]. Human CRC tissues lack decorin expression in vivo and in vitro [38], and adenovirus-mediated transduction of decorin into human colon cancer cell lines significantly reduces their colony-forming ability [38]. Loss of decorin favors intestinal tumor formation in mice, whereas enhancement of decorin production inhibited the proliferation and migration of CRC cells and promoted apoptosis in parallel with upregulated E-cadherin expression [40,43]. In line with that, oncolytic adenovirus-mediated decorin and GM-CSF gene transfer inhibited tumor growth in a colorectal tumor model [44]. The tumor suppressor effect of decorin in CRC was shown to be mediated via arrest of the cell cycle in G1 phase [26].

Several studies have addressed the role of decorin in human metastatic cancers, and results are contradictory. Cawthorn and coworkers found that high decorin expression was associated with lymph node metastasis and poor clinical outcome in breast cancer; thus, decorin was proposed as an adverse prognostic and predictive biomarker [45]. Conversely, downregulation of decorin and lymph node metastasis independently predicted poor prognosis in oral squamous cell carcinomas [46]; DCN expression in osteosarcoma cells blocked their metastatic spread to the lungs [47]; and virus-mediated decorin delivery inhibited the growth of breast cancer and suppressed metastasis formation in various organs [48,49,50,51].

Published data about the effect of decorin on hepatic metastasis of CRC are, however, lacking. Thus, we have collected archive biopsy samples of patients with liver metastases of colorectal cancer, and built tissue microarrays containing samples of liver metastasis, surrounding non-tumorous liver, primary tumor and normal colon from each patient. The highest decorin expression was found in normal colon, but decorin was also abundant in the primary tumor stroma. However, the liver metastasis of the same tumor often displayed reduced amounts of decorin, and in a greater extent in the more aggressive grade III tumors, and in metastases with replacement growing pattern (Figure 1). These observations suggest that the decreased decorin expression in liver metastasis of CRC may correlate with the aggressiveness of the tumor.

As fibroblasts are the main producers of decorin, we tested whether colon cancer cells can influence decorin expression of liver fibroblast cells in a model that mimicked tumor cell invasion to the liver. LX2 human stellate cells, when treated with the CM of HT29 CRC cells, displayed significantly reduced DCN expression (Figure 2). Earlier we found that the CM of hepatoma cells inhibited decorin production of the LX2 stellate cell line [33]; here we show evidence that tumor cells of a different tissue origin can also suppress decorin production by liver fibroblasts. These data, together with our observations in human CRC tissue samples, are compatible with an anti-tumor role for decorin in CRC and its liver metastases.

Our findings are in accordance with earlier reports where adenovirus-mediated decorin gene delivery inhibited the proliferation of colon and squamous carcinoma [52], urothelial malignancies [39], prostate cancer [53], breast cancer bone metastases [51] and pancreatic tumor [54].

Decorin delivery effectively downregulated the activity of EGFR, PDGFR and HGF/MSPR receptors (Figure 5), all known targets of decorin [23,27,32]. As a consequence, the activities of downstream signaling pathways such as RAS/MEK/ERK, Akt/mTOR/S6K, and PLCγ were also attenuated (Figure 7). Decorin increased the level of AMPK, a protein involved in multiple cellular processes such as cell growth, autophagy and metabolism [55]. Decorin was previously shown to stimulate AMPK-related pro-autophagy pathways in parallel with suppressing the anti-autophagic PI3K/Akt/mTOR/p70S6K pathway [56,57]. Hence we hypothesize that AMPK may be responsible for the enhanced function of mTORC2 complex indicated by the increase of Akt phosphorylation at the Ser473 residue [58]. Enhancement of AMPK function is further amplified by the decreased level of its inhibitor, the anti-autophagic WNK1 [59]. Furthermore, WNK1 is phosphorylated by Akt as a feed-back loop [60]. LKB1 is one of the main stimulators of AMPK [55]. Although LKB1 was not measured in our study, ERK1/2 is known to exert a blocking effect on the tumor suppressor LKB1 [61]. Thus, decorin-mediated inhibition of ERK signaling may set LKB1 free to activate AMPK. Based on these observations, we speculate that decorin may promote autophagy in our model system; however, further studies are needed to confirm this assumption. LKB1/AMPK/mTOR signaling is the master regulator of cellular metabolism, and, along with MAP kinases, controls metabolic reprogramming [61,62]. Therefore, decorin presumably plays a crucial role in counteracting the Warburg-effect in the metabolism of cancer cells, but further clarification and discussion remains for future studies.

Decorin delivery led to enhanced activity of the p38 MAPK/MSK/CREB axis in tumor-bearing livers. This pathway is known to provoke cell cycle arrest at both the G1/S and G2/M checkpoints [63]. As a mechanism of action, p38 and CREB directly phosphorylate and recruit the p53 tumor suppressor, lending enhanced stability to the protein [64,65]. p53 action is further strengthened by AMPK/LKB1 phosphorylation [66,67]. Although the extracellular signals and receptors involved in the activation of the p38 pathway have not been identified in our model, downstream events point to cell cycle arrest in a p53-dependent manner. Inhibition of STAT and c-Jun proteins also corroborate the antitumor properties of decorin. Besides being regulated by cytokine receptors, STATs are also downstream of RTKs; thus, inhibition of RTKs by decorin may be responsible for decreased STAT levels [68]. A full discussion of the implications of decorin in inflammation and immune responses is beyond the scope of the present manuscript. Regarding the proto-oncogenic c-Jun, we found increased levels of JNKs, the main activators of the c-Jun protein. However, we hypothesize that the marked decrease in phospho-c-Jun upon decorin transfection results from decreased activity of ERK1/2, a known upstream activator of c-Jun [69,70]. Apart from its action on c-Jun, JNK signaling stabilizes and activates p53, and induces apoptosis [71], which is in line with our observations described above. We saw relatively small changes in signaling proteins in the metastasis-bearing livers of decorin-overexpressing mice, supposedly due to the much lower tumor burden of decorin-delivered mice. Despite that, we think our results can highlight the hallmark signaling events affected by decorin in this model.

In CRC, various treatment approaches such as surgery, radiation therapy, chemotherapy, immunotherapy, and targeted therapy are applied [72]. Patients with colon cancer were among the first major beneficiaries of the introduction of targeted therapy, mainly because of the well-known molecular mechanisms responsible for the malignant phenotype [72]. At present, EGFR inhibitors such as cetuximab (Erbitux) and panitumumab (Vectibix) are widely used in the targeted therapy of CRC [73]. Decorin is known to effectively block the activity of EGFR but also of VEGFR, PDGFR, Met and Raf kinases. Thus, decorin either alone or as an adjuvant could have a place in the clinical treatment of CRC.

## 5. Conclusions

The tumor microenvironment plays a determining role in cancer development by regulating multiple processes between the ECM and tumor cells. Decorin, a prototype member of the SLRP family, has gained recognition for its essential roles in multiple pathologies including cancer. Studies on mice with ablated decorin genes revealed that the lack of decorin is permissive for tumor development. Concordantly, reduced expression of decorin was observed in several types of cancer, suggesting that decorin tends to act as a tumor suppressor in these contexts. Moreover, when applied as a therapeutic agent, decorin effectively inhibited tumor formation, progression, angiogenesis, and metastasis in a multitude of experimental models. In the present study, we report that liver metastases of CRC display reduced amounts of decorin when compared to the primary tumor. Colon carcinoma tumor cells in vitro were able to suppress decorin production of LX2 stellate cells, a fibroblast cell line of liver origin. Moreover, liver-targeted decorin delivery effectively inhibited metastasis formation of colon cancer. When investigating the mechanism of action, we saw that overexpressed decorin was able to reduce the activity of multiple RTKs including EGFR, an important player in CRC pathogenesis. Downstream of that, we observed weakened signaling of ERK1/2, PLCγ, Akt/mTOR, STAT and c-Jun pathways, while p38 MAPK/MSK/CREB and AMPK were upregulated, culminating in enhanced p53 function. Therefore, decorin as “a guardian from the matrix” may be an invaluable tool in combatting colorectal cancer.

## Figures and Tables

**Table 1:** Changes in signaling proteins detected by phosphokinase array. The table shows the relative protein levels in decorin-transfected (DCN) versus null-vector-transfected mice inoculated with c38 colorectal cancer cells. Data represent the mean of two independent experiments.

Protein	Phosphorylation Site	Relative Level in DCN Group (%)	*p* Value
Akt 1/2/3 (S473)	S473	139.277	0.085
Akt 1/2/3 (T308)	T308	96.147	0.083
AMPKα1 (T183)	T183	107.259	0.027
AMPKα2 (T172)	T172	109.825	0.124
Chk-2	T68	105.490	0.517
c-Jun	S63	80.634	0.016
CREB	S133	114.239	0.103
EGF R	Y1086	97.511	0.274
eNOS	S1177	96.573	0.528
ERK1/2	T202/Y204, T185/Y187	79.370	0.045
FAK	Y397	87.540	0.343
Fgr	Y412	73.382	0.047
Fyn	Y420	99.185	0.220
GSK-3α/β	S21/S9	98.895	0.126
Hck	Y411	103.026	0.210
HSP27	S78/S82	85.398	0.014
HSP60	-	107.067	0.042
JNK 1/2/3	T183/Y185, T221/Y223	114.402	0.438
Lck	Y394	100.697	0.567
Lyn	Y397	109.082	0.371
MSK1/2	S376/S360	117.438	0.038
p27	T198	106.962	0.336
p38α	T180/Y182	108.060	0.435
p53 (S46)	S46	104.783	0.276
p53 (S15)	S15	112.702	0.185
p53 (S392)	S392	117.981	0.064
p70 S6 Kinase	T421/S424	79.221	0.050
p70 S6 Kinase (T389)	T389	78.876	0.059
PDGF Rβ	Y751	70.647	0.012
PLC-γ1	Y783	75.498	0.026
PRAS40	T246	116.635	0.067
PYK2	Y402	79.806	0.083
RSK1/2/3	S380/S386/S377	90.656	0.072
Src	Y419	110.985	0.089
STAT2	Y689	87.833	0.139
STAT3	Y705	84.852	0.117
STAT3	S727	96.977	0.462
STAT5a	Y694	85.820	0.157
STAT5a/b	Y694/Y699	82.277	0.287
STAT5b	Y699	75.547	0.016
STAT6	Y641	92.892	0.083
TOR	S2448	97.492	0.346
WNK1	T60	82.123	0.053
Yes	Y426	97.223	0.313
β-Catenin	-	84.948	0.025

## Supplementary Materials

The following are available online at https://www.mdpi.com/2218-273X/10/8/1199/s1, Table S1: List of biopsy samples of CRC with liver metastasis, Table S2: Scoring results of decorin immunostaining on TMA slides. Figure S1: Correlation between SMA and decorin staining intensities in surrounding liver samples of CRC metastases.
