# Supplementary material for: The Protective Role of Decorin in Hepatic Metastasis of Colorectal Carcinoma
Source: Biomolecules. 2020 Aug 18;10(8):1199. doi: 10.3390/biom10081199 (PMC7465536; doi:10.3390/biom10081199)
Supplement: Supplementary file 1 [file biomolecules-10-01199-s001.pdf]

Supplementary Table S1. List of biopsy samples of CRC with liver metastasis.

Patient ID	Sex	Age	Grade:	T:	N:	M:	Stromal reaction in PT *	Growth pattern in LM **
1	female	61	II	3	1	x	Intermediate	Replacement
2	female	45	I	3	1	x	Mature	Desmoplastic
3	male	64	II	3	1	1	Immature	Desmoplastic
4	female	46	II	3	0	x	Mature	Desmoplastic
5	male	48	II	4	2	x	Immature	Replacement
6	female	66	II	4	1	x	Mature	Desmoplastic
7	male	69	II	3	0	x	Mature	Desmoplastic
8	male	68	II	3	2	x	Mature	Replacement
9	male	52	II	4	1	1	Mature	Desmoplastic
10	female	56	II	3	0	x	Mature	Desmoplastic
11	female	73	II	3	0	x	Mature	Desmoplastic
12	female	69	II	3	0	2	Mature	Replacement
13	male	69	II	3	2	1	Immature	Replacement
14	female	66	II	3	1	x	Mature	Desmoplastic
15	female	53	II	1	0	x	Intermediate	Replacement
16	female	68	II	3	2	x	Immature	Desmoplastic
17	male	67	III	3	2a	x	Mature	Replacement
18	female	35	II	3	2a	x	Intermediate	Desmoplastic
19	male	66	II	2a	0	x	Intermediate	Desmoplastic
20	male	66	III	3	2a	1	Intermediate	Replacement
21	male	59	II	3	1	1	Mature	Desmoplastic
22	male	49	III	3	1	1	Mature	Desmoplastic
23	male	51	III	3	1	1	Mature	Replacement
24	female	61	II	4a	2b	1	Mature	Replacement
25	male	76	III	3	x	x	Mature	Desmoplastic
26	male	57	III	3	1	x	Intermediate	Desmoplastic
27	male	43	II	1	1	x	Mature	Desmoplastic
28	female	59	II	2	0	x	Mature	Desmoplastic
29	female	70	II	3	0	1	Mature	Desmoplastic
30	male	56	II	3	0	x	Mature	Desmoplastic

Abbreviations: PT: primary tumor, LM: liver metastasis.

*Categorization as described by Ueno et al. Ann Surg Oncol (2015) 22:1504–1512

** Categorization based on Höppener et al. Clinical & Experimental Metastasis (2019) 36:311–319

Supplementary Table S2. Scoring results of decorin immunostaining on TMA slides.

ID	Normal Average	Primer Average	Metastasis Average	SL Average
**1**	50,14	34,36	49,14	52,07
**2**	139,00	23,60	16,48	23,10
**3**	44,00	68,85	25,41	88,34
**4**	30,05	85,29	60,91	14,99
**5**	147,82	117,93	29,95	38,68
**6**	188,57	97,89	0,52	18,51
**7**	41,05	94,32	17,97	27,45
**8**	89,87	37,64	10,13	6,93
**9**	104,82	33,57	63,74	37,89
**10**	96,74	107,53	31,18	61,25
**11**	141,67	81,25	17,04	114,37
**12**	56,66	20,33	24,22	106,26
**13**	56,58	53,60	40,21	12,01
**14**	72,15	142,65	87,35	32,19
**15**	23,05	26,14	31,46	12,44
**16**	179,67	118,48	130,44	116,08
**17**	101,71	138,33	65,67	61,88
**18**	158,52	62,53	122,16	3,95
**19**	134,90	83,07	77,92	48,82
**20**	121,20	138,81	22,67	75,26
**21**	199,89	201,10	71,82	26,64
**22**	142,47	121,04	9,31	71,06
**23**	54,96	28,06	55,75	29,39
**24**	129,62	124,62	1,54	70,77
**25**	84,20	39,87	7,06	20,76
**26**	3,71	10,18	34,26	6,86
**27**	101,64	83,69	29,17	5,68
**28**	79,30	25,58	32,77	33,17
**29**	105,84	52,44	65,51	26,15
**30**	135,89	136,74	77,63	36,13

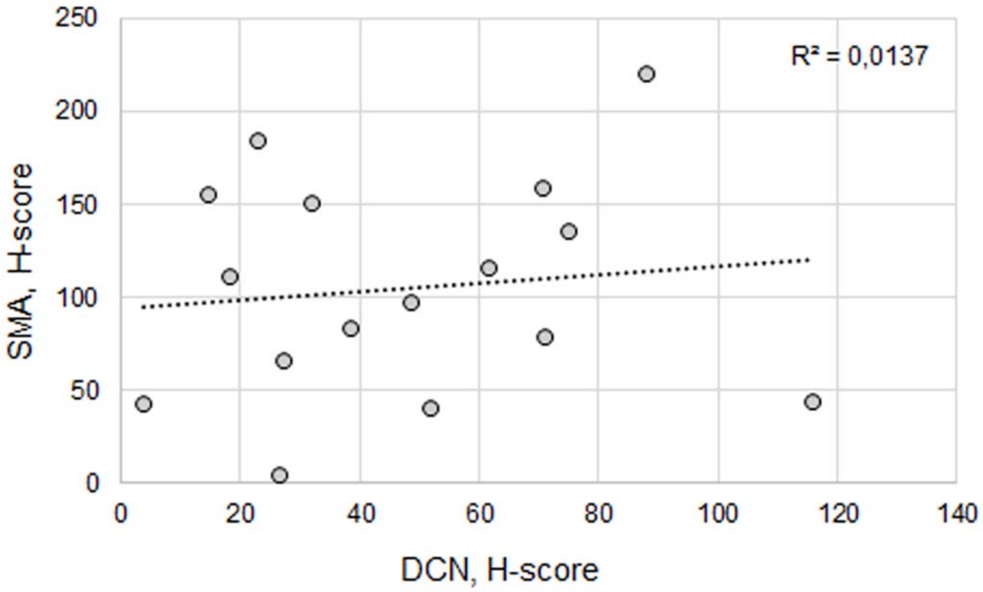

Supplementary Figure S1. Correlation between SMA and decorin staining intensities in surrounding liver samples of CRC metastases. Decorin score is independent from SMA score meaning that decorin content of liver samples are not proportional with its stroma abundance.
